# Long-term outcomes of coronary artery bypass grafting versus stent-PCI for unprotected left main disease: a meta-analysis

**DOI:** 10.1186/s12872-017-0664-5

**Published:** 2017-09-06

**Authors:** Salvatore De Rosa, Alberto Polimeni, Jolanda Sabatino, Ciro Indolfi

**Affiliations:** 1 Division of Cardiology, Department of Medical and Surgical Sciences, “Magna Graecia” University, 88100 Catanzaro, Italy; 2 URT-CNR, Department of Medicine, Consiglio Nazionale delle Ricerche (CNR), 88100 Catanzaro, Italy

**Keywords:** LMCA, CABG, PCI

## Abstract

**Background:**

Coronary artery bypass graft (CABG) surgery has traditionally represented the standard of care for left main coronary artery (LMCA) disease. However, percutaneous coronary intervention with stent implantation (PCI) has more recently emerged as a valuable alternative. The long-time awaited results of the largest randomized trials on the long-term impact of PCI versus CABG in LMCA disease, the newly published NOBLE and EXCEL studies, revealed contrasting results. Thus, aim of the present meta-analysis was to review the most robust evidence from randomized comparisons of CABG versus PCI for revascularization of LMCA.

**Methods:**

Randomized studies comparing long-term clinical outcomes of CABG or Stent-PCI for the treatment of LMCA disease were searched for in PubMed, the Chochrane Library and Scopus electronic databases. A total of 5 randomized studies were selected, including 4499 patients.

**Results:**

No significant difference between CABG and PCI was found in the primary analysis on the composite endpoint of death, stroke and myocardial infarction (OR = 1·06 95% CI 0·80–1·40; *p* = 0·70). Similarly, no differences were observed between CABG and PCI for all-cause death (OR = 1·03 95% CI 0·81–1·32; *p* = 0·81). Although not statistically significant, a lower rate of stroke was registered in the PCI arm (OR = 0·86; *p* = 0·67), while a lower rate of myocardial infarction was found in the CABG arm (OR = 1·43; *p* = 0·17). On the contrary, a significantly higher rate of repeat revascularization was registered in the PCI arm (OR = 1·76 95% CI 1·45–2·13; *p* < 0·001).

**Conclusions:**

The present meta-analysis, the most comprehensive and updated to date, including 5 randomized studies and 4499 patients, demonstrates no difference between Stent-PCI and CABG for the treatment of LMCA disease in the composite endpoint of death, stroke and myocardial infarction. Hence, a large part of patients with unprotected left main coronary artery disease can be managed equally well by means of both these revascularization strategies.

**Electronic supplementary material:**

The online version of this article (doi:10.1186/s12872-017-0664-5) contains supplementary material, which is available to authorized users.

## Background

Significant left main coronary artery (LMCA) disease, occurring in up to 10% of patients undergoing coronary angiography, is a life-shortening and disabling condition [1]. Myocardial revascularization with coronary artery bypass graft (CABG) surgery has represented the standard of care for patients with significant LMCA disease [2]. However, percutaneous coronary intervention with stent implantation (PCI) has emerged as a valuable alternative, given the lower peri-procedural risk and the continuous improvement in device technology, associated with better procedural performance and long-term clinical prognosis. In this scenario, the most recent European guidelines for myocardial revascularization endorse PCI with a class I recommendation for LMCA disease with a SYNTAX score (SS) < 22 and and with a class IIa recommendation patients with a SS between 23 and 32 [2]. A previous meta-analysis found no significant differences in the composite endpoint of death, stroke and myocardial infarction, between PCI and CABG, with a significantly higher rate of repeated revascularization in the PCI arm and a higher incidence of stroke in CABG-treated patients [3]. In this context, PCI has been used in a growing percentage of cases in the last years, especially in low- and intermediate-risk patients [4, 5]. However, only two randomized trials were available until recent times, strongly limiting the validity of previous meta-analyses when most of the evidence was coming from non-randomized studies [6]. On the contrary, a larger body of evidence is currently available on the long-term clinical impact of PCI versus CABG in LMCA disease, including the largest, newly published, NOBLE and EXCEL trials, yielding opposite results [7, 8]. Hence, the aim of the present meta-analysis was to evaluate the long-term outcome of Stent-PCI and CABG for the treatment of LMCA stenoses, on the basis of the larger clinical evidence available to date.

## Methods

### Search strategy and study selection

Published randomized trials comparing percutaneous to surgical revascularization of left main coronary stenoses were searched for within PubMed, Cochrane and Scopus electronic databases up to October 31th 2016. The following key words were used for the search: “left main”, “percutaneous coronary intervention” and “coronary artery bypass graft”. Time of publication and language were not limiting criteria for our analysis. All reports including the search terms were independently screened by two investigators for relevance and eligibility. Additionally, references from relevant articles were also scanned for eligible studies. The authors discussed their evaluation and any disagreement was resolved through discussion and re-reading. All selected trials were thoroughly checked and classified by author’s institution in order to avoid any effect from duplicity of data.

Studies were considered eligible if the following statements were applying: a) they involved a study population with LMCA disease; b) they compared PCI with coronary artery stenting versus CABG; c) follow-up length ≥ 3 years; d) they reported clinical outcome data (death, MACCE, AMI, revascularization, stroke). Exclusion criteria were (just one was sufficient for study exclusion): duplicate publication, pre-specified endpoint measure not specified. Studies reporting only lesion-based analyses were excluded from the present work.

### Data abstraction, validity assessment and analysis

Baseline characteristics, as well as numbers of events, were extracted from the single studies, through carefully scanning of the full article by two independent reviewers (AP, JS). Divergences were resolved by consensus. In particular, the following data were abstracted: year of publication, location, number of study patients, study design, clinical outcome data (death, MACCE, AMI, revascularization, stroke), baseline patients’ characteristics. Selection and data abstraction was performed according to the PRISMA statement (Additional file 1: Supplementary material 1) [9]. The primary analysis was based on the composite endpoint of death, myocardial infarction and stroke. In addition, results on the composite endpoint including death, myocardial infarction, stroke and repeat revascularization were also analyzed. Furthermore, meta-analysis results of single endpoints are also provided.

### Statistical analysis

The summary measure used was the Odds Ratio (OR) with 95% interval confidence. A random-effects model was used, as previously described, to combine the collected values [10]. This model calculates a weighted average of the relative risks by incorporating within-study and between-study variations. Heterogeneity was assessed by means of the Cochrane Q test using a chi-squared function, with *p* values <0·10 considered significant for heterogeneity, as previously described [11]. Additionally, I^2^ values were calculated for estimation of variation in weighted mean differences among studies attributable to heterogeneity. Any I^2^ value >20% was considered significant. Small study effects were evaluated through graphical inspection of funnel plots, as already previously described [12]. Forest plots were used to graphically display the results of the meta-analysis, as already previously described [13]. Briefly, the measure of effect (OR) for each single study included (represented by a square) is plotted, together with confidence intervals, represented by horizontal lines. The area of each square is proportional to the study’s weight in the meta-analysis. The overall measure of effect is reported on the bottom line of the plot as a diamond, whose lateral ends indicate the confidence interval for the summary effect. Meta-regression analyses were calculated using the unrestricted maximum likelihood model, as previously described [14]. Results of meta-regression are graphically depicted plotting the logarithm of the OR against a moderator variable, where each study is represented by a circle (effect size) and the area of each circle is proportional to the weight of that study in the analysis, as previously reported [15]. Analyses were performed by means of RevMan 5·3 and Open Meta Analyst.

## Results

### Search results

**Fig. 1:**
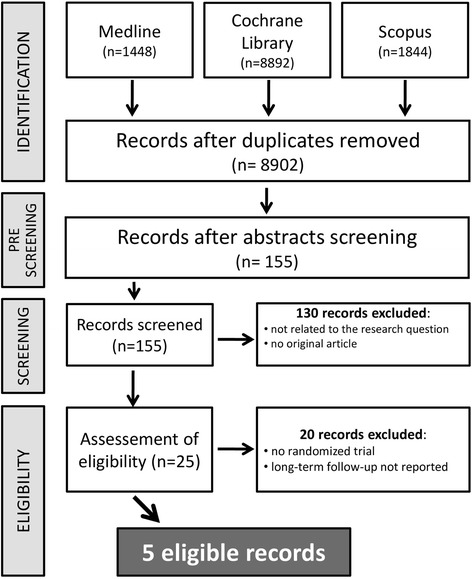
Study selection flow chart

Our search retrieved a total of 8902 entries, which were reduced to 155 studies after an initial pre-screening. 130 studies were then excluded for one of the following reasons: a) they were not related to our research question b) they weren’t original articles. In the assessment of eligibility further 20 studies were excluded. Finally, a total of 5 studies were available for the analysis including 4499 patients [7, 8, 16–18]. The study selection procedure is reported in details in Fig. 1.

### Study characteristics

Only multicenter, randomized, trials were included in the present meta-analysis. Table 1 summarizes the most relevant characteristics of the selected studies. Not surprisingly, quality assessment revealed a high study quality (Additional file 2: Figure S1). Of course, the specific study designs made both patients’ and investigators’ blinding impossible. Nevertheless, endpoint assessment and data analysis was blinded in all included studies.

**Table 1:** Characteristics and Endpoint definitions of included randomized trials

Study	Year	Location	N	Study design	Primary endpoint	Mortality reported	Type of stent	MACCE definition	Follow up (years)
EXCEL [7]	2016	Multicenter	1905	RCT	death, MI, Stroke,	Yes	DES (EES)	death, MI, Stroke, IDR	3
NOBLE [8]	2016	Multicenter	1184	RCT	death, MI, Stroke, RR	Yes	DES (SES/BES)	death, MI, Stroke, RR	5
LE MANS [18]	2016	Multicenter	105	RCT	LVEF	Yes	DES/BMS	death, MI, Stroke, TVR	10
PRECOMBAT [17]	2015	Multicenter	600	RCT	death, MI, Stroke, IDR	Yes	DES (SES)	death, MI, Stroke, IDR	5
SYNTAX [16]	2014	Multicenter	705	RCT	death, MI, Stroke, RR	Yes	DES (PES)	death, MI, Stroke, RR	5

*Abbreviations: MI* myocardial infarction, *IDR* Ischemia driven revascularization, *RR* repeat revascularization, *LVEF* left ventricle ejection fraction, *TVR* target vessel revascularization, *RCT* randomized clinical trials (see Additional file 5)

Across the studies, patients were predominantly male and approximately one fourth of patients had diabetes mellitus. Although prevalence of single cardiovascular risk factors was not equal among the studies, treatment arms were generally well balanced. More details on patients’ characteristics are provided in Table 2. Previous studies used 1st generation paclitaxel- [16], sirolimus-eluting DES [17], or both PES and SES were used in 35% of the LMCA PCIs performed within the LE MANS study, whereas a BMS was used in the remaining cases, due to a larger diameter of the LMCA [18]. On the contrary, the newly published trials mostly used 2nd generation DES (89·1% in the NOBLE, 100% in the EXCEL) [7, 8]. In fact, 1st generation sirolimus–eluting stents were only used in 42 patients (10·9%) in the NOBLE with the remaining patients treated with bioresorbable polymer biolimus-eluting stents, while only 2nd generation everolimus-eluting stents were used in the EXCEL.

**Table 2:** Baseline patient’s characteristics

	SYNTAX 2014 [16]		PRECOMBAT 2015 [17]		LE MANS 2016 [18]		NOBLE 2016 [8]		EXCEL 2016 [7]	
	PCI	CABG	PCI	CABG	PCI	CABG	PCI	CABG	PCI	CABG
N of patients, *n*	357	348	300	300	52	53	592	592	948	957
Age, yrs.	65,4	65,6	61,8	62,7	60,6	61,3	66,2	66,2	66	65,9
Male, %	72	76	76	77	60	73	80	76	76,2	77,5
Hypertension, %	67	62	54	51	75	70	65	66	74,5	73,9
Dyslipidaemia, %	81	75	42	40	65	60	82	78	71,5	69,3
Diabetes, %	24	26	34	30	19	17	15	15	30,2	28
Ejection fraction, %	59,6	58,7	61,7	60,6	53,5	53,7	60	60	57	57,3
Hospitalization, days	6,1	13,6	3,1	8,4	6,8	12,04	2	9	5,4	12,7

*yrs* years, *PCI* percutaneous coronary intervention, *CABG* coronary artery bypass grafting

Intracoronary imaging can be particularly useful during PCI of the LMCA. In fact, it was largely used in the randomized studies included in the present analysis, although in different proportions of patients. In fact, while PCI guidance by means of intravascular ultrasound was used in about 80% of patients in the EXCEL and in 91·2% of cases in the PRECOMBAT [7, 17], it was used in a lower number of cases in the NOBLE (47%) [8], while it was used only for assessment of post-PCI result in the LE MANS study [18]. Finally, use of intracoronary imaging to guide PCI was left at operator’s discretion in the SYNTAX study, but no information has been reported on the actual use in patients treated with PCI for LMCA disease [16, 19].

The range of LMCA diameters eligible for enrollment were not homogeneus across the studies, reflecting the different availability of stents at different times. In fact, while LMCA diameters up to 4·25 mm were allowed to be randomized in the contemporary EXCEL study [7], only LMCAs with a reference diameter < 3·8 mm could be treated in the LE MANS, with BMS used in the remaining 65% of cases [18]. Unfortunately, specific diameter boundaries for eligibility of LMCA disease are not precisely reported in the remaining studies.

### Meta-analysis results

**Fig. 2:**
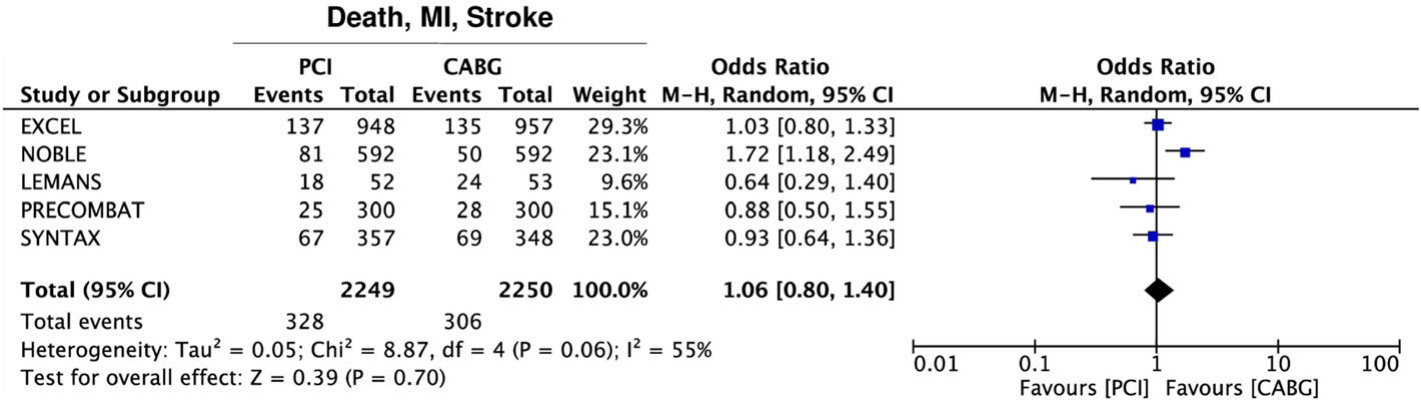
Meta-analysis of the composite endpoint of death, myocardial infarction (MI) and stroke. Forest plot and summary effect of the difference in the incidence of the composite endpoint of death, myocardial infarction (MI) and stroke, showing no difference between CABG and PCI

**Fig. 3:**
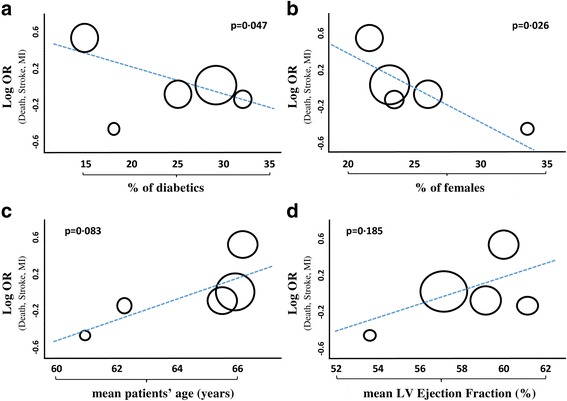
Metaregression-analysis on the composite endpoint of death, myocardial infarction (MI) and stroke. Panel **a** Meta-regression analysis of all included studies, showing a significant inverse interaction of the effect size with the proportion of diabetics included in single studies, indicating that CABG performed better than PCI in studies that included a larger number of diabetics. Panel **b** Meta-regression analysis of all included studies, showing a significant inverse interaction of the effect size with the proportion of female patients included in single studies, indicating that CABG performed better than PCI in studies that included a larger number of females. Panel **c** Meta-regression analysis of all included studies, showing a non-significant direct interaction of the effect size with mean patients’ age from single studies, suggesting that PCI may perform better than CABG in older patients. Panel **d** Meta-regression analysis of all included studies, showing a non-significant direct interaction of the effect size with mean left ventricular ejection fraction from single studies. Each study is represented by a circle that shows the effect size. The area of each circle is proportional to that study’s weight in the analysis

The primary analysis, summing up all results of the 5 randomized trials included on the composite endpoint of death, myocardial infarction and stroke (Fig. 2), revealed no difference between the CABG- and the PCI-treatment arms (OR = 1·06 95% CI 0·80–1·40; *p* = 0·70). No evidence of funnel plot asymmetry was found for this endpoint (Additional file 3: Figure S2A). At meta-regression analysis, a significant inverse relationship was found with the percentage of diabetics (*p* = 0·047) (Fig. 3a) or female patients included in the study (*p* = 0·026) (Fig. 3b), suggesting that CABG performs better in diabetics and female patients. On the other hand, although not statistically significant, a direct relationship was evident with mean patients’ age (*p* = 0·083) (Fig. 3c) and mean left ventricular ejection fraction (*p* = 0·185) (Fig. 3d), suggesting that Stent-PCI performs better than CABG with increasing patients’ age or with worse cardiac function. On the contrary, no interaction was observed with the proportion of ACS patients enrolled into single studies (*p* = 0·953). Despite the studies included have different follow up lenghts, this was shown to have no substantial effect on meta-analysis results (Additional file 4: Figure S3).

**Fig. 4:**
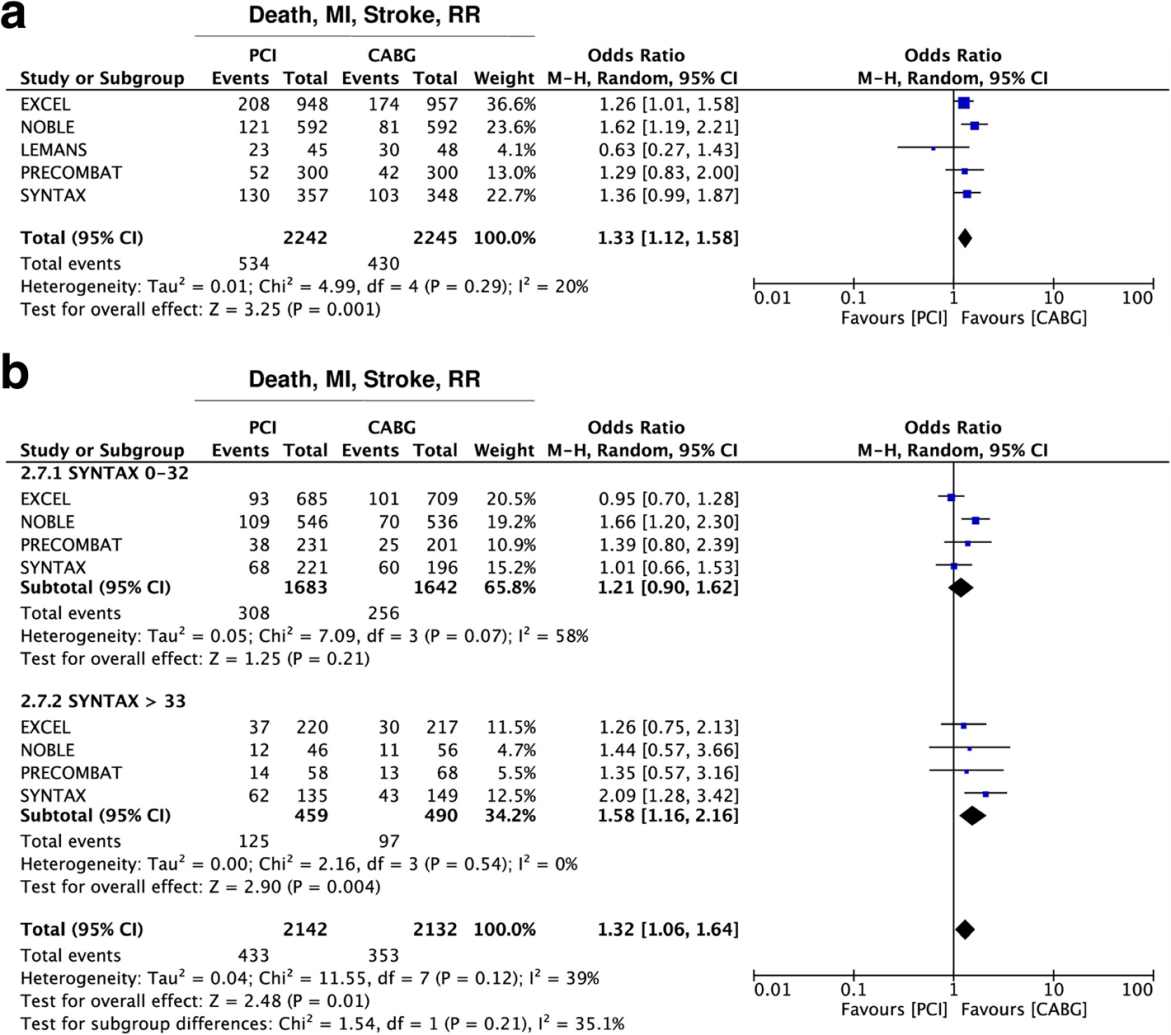
Meta-analysis of the composite endpoint of death, myocardial infarction (MI), stroke and repeat revascularization (RR). Panel **a** Forest plot and summary effect of the difference in the incidence of the composite endpoint of death, MI, stroke and RR, showing a significantly lower incidence in the CABG arm. Panel **b** Subgroup analysis showing no difference between CABG and PCI within the lower tertiles of the SYNTAX Score (SYNTAX 0–32), but better performance of CABG within the highest tertile (SYNTAX >33)

Addition of repeat revascularization (RR) to the composite endpoint (death, myocardial infarction, stroke and repeat revascularization) revealed a better performance of CABG over Stent-PCI (OR = 1·33 95% CI 1·12–1·58; *p* = 0·001) (Fig. 4a). However, patients’ stratification on the basis of the SS showed that the better performance of CABG on this endpoint was indeed limited to the higher risk tertile of the SYNTAX score (SS > 32) (OR = 1·58 95% CI 1·16–2·16; *p* = 0·004), with no difference between the two treatment arms for the low- (SS ≤ 22) to intermediate-risk (SS in the 22–32 range) tertiles (OR = 1·21 95% CI 0·90–1·62; *p* = 0·21) (Fig. 4b). No evidence of funnel plot asymmetry was found for this endpoint (Additional file 3: Figure S2B).

**Fig. 5:**
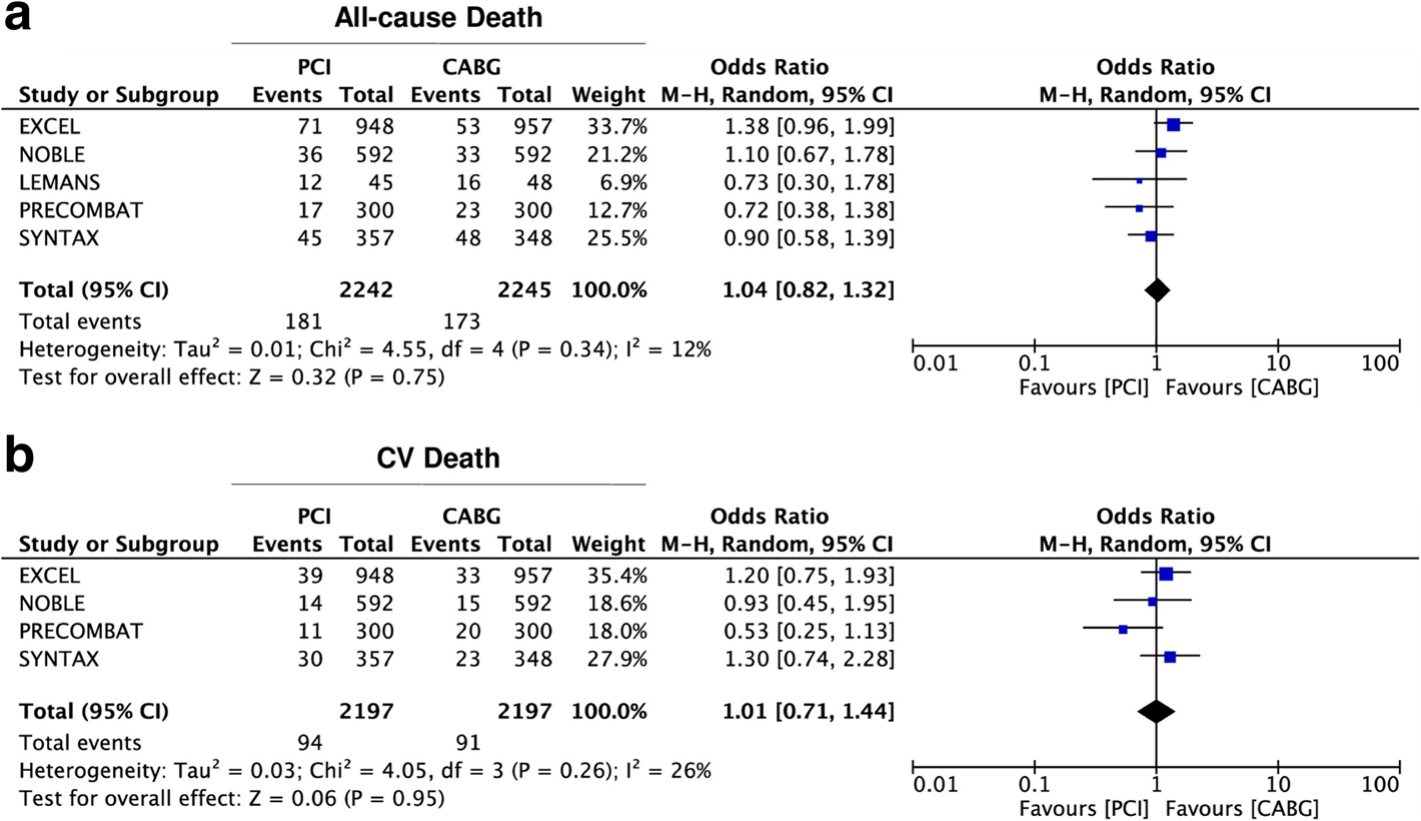
Meta-analysis of death and cardiovascular (CV) death. Panel **a** Forest plot and summary effect of the difference in the incidence of death, showing no difference between CABG and PCI. Panel **b** Forest plot and summary effect of the difference in the incidence of CV death, showing no difference between CABG and PCI

**Fig. 6:**
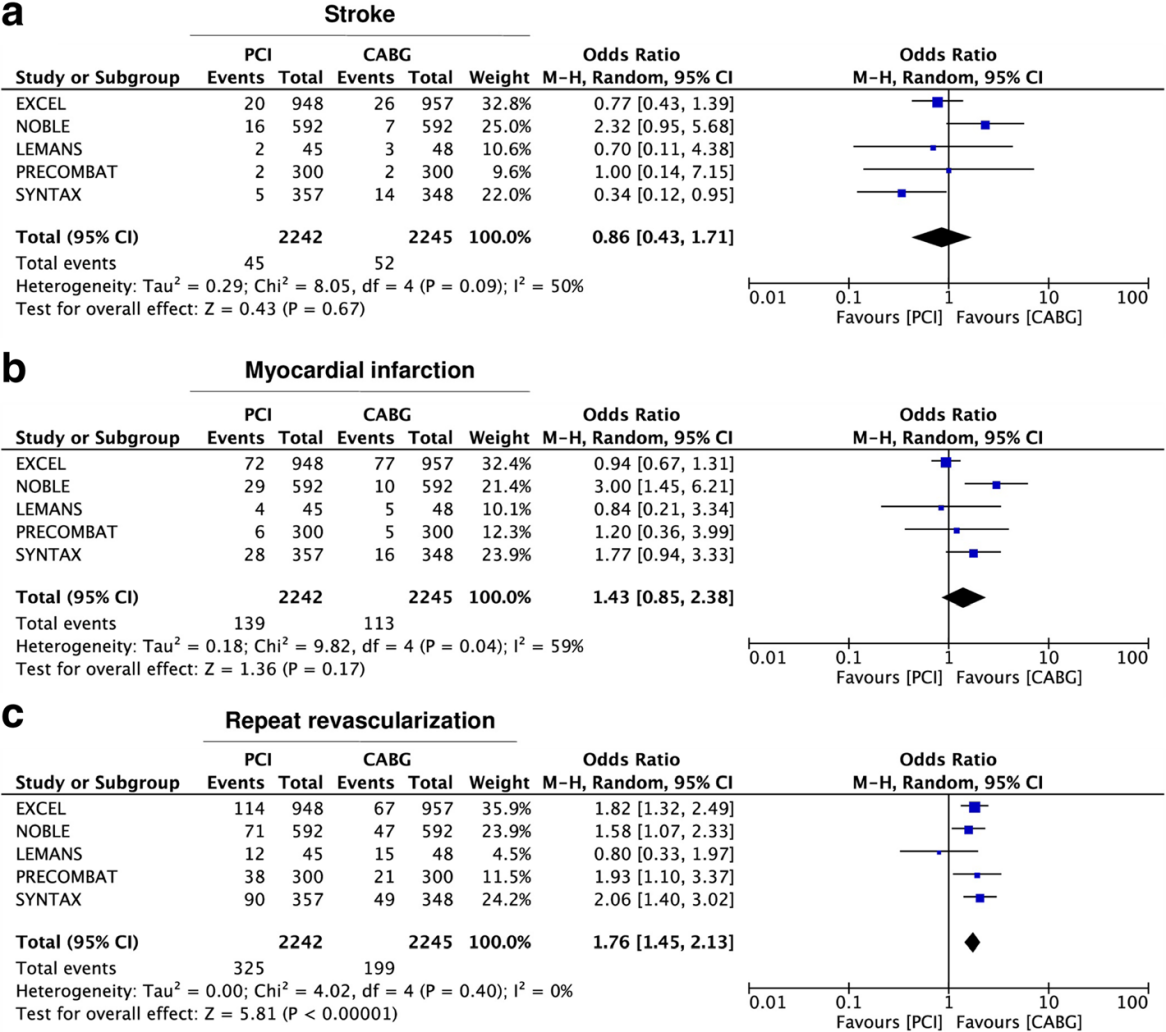
Meta-analysis of single clinical endpoints. Panel **a** Forest plot and summary effect of the difference in the incidence of stroke, showing no difference between CABG and PCI. Panel **b** Forest plot and summary effect of the difference in the incidence of myocardial infarction (MI), showing no significant difference between CABG and PCI. Panel **c** Forest plot and summary effect of the difference in the incidence of repeat revascularization (RR), showing a significantly lower incidence in the CABG arm

Analyzing results for single endpoints, we found no difference betwen CABG and PCI in terms of all-cause death (OR = 1·04 95% CI 0·82–1·32; *p* = 0·81) (Fig. 5a) and cardiovascular death (OR = 1·01 95% CI 0·71–1·44; *p* = 0·95) (Fig. 5b). On the other hand, a trend towards a lower rate of stroke was registered in the Stent-PCI arm (OR = 0·86 95% CI 0·43–1·17; *p* = 0·67), altough not statistically significant (Fig. 6a). Similarly, a lower rate of myocardial infarction (MI) was found in the CABG arm (OR = 1·43 95% CI 0·85–2·38; *p* = 0·17), altough not statistically significant (Fig. 6b). On the contrary, a significantly higher rate of repeat revascularization was registered in the Stent-PCI arm (OR = 1·76 95% CI 1·45–2·13; *p* < 0·001) (Fig. 6c).

## Discussion

This is the most comprehensive and updated meta-analysis of randomized studies comparing the long-term outcome after treatment of LMCA disease with CABG or Stent-PCI. Summing up the best clinical evidence available to date, including 5 randomized studies and 4499 patients, no difference was found between Stent-PCI and CABG for the treatment of LMCA disease, for the composite endpoint of death, stroke and myocardial infarction. Interestingly, results on this endpoint were quite homogeneous across individual studies, with the exception of the NOBLE trial, where this endpoint was actually driven by an excess of myocardial infarction and stroke registered in the PCI-treated group, which could be related, at least in part, to some specific characteristics of that study [8]. In fact, the higher stroke rate registered in the PCI arm of the NOBLE trial, which was higher compared to the CABG arm is a black swan. This phenomenon was mainly related to events occurred after interruption of dual antiplatelet therapy (DAPT), 1 year after the index PCI [8]. On the other hand, DAPT was still ongoing in over 70% of patients at 2 years and in more than 65% of patients at 3 years in the EXCEL study, where the rate of stroke and MI in the PCI arm was lower compared to that registered among CABG-treated patients [7]. These relevant differences between the studies suggest that prologation of DAPT, as well as the use of newer P2Y12 antagonists could have contributed to the better performance of PCI in the EXCEL trial, and could help the further improvemet of the clinical outcome after PCI of the LMCA. In fact, amog the two more recent RCTs, prasugrel or ticagrelor were used in only 26·9% of patients treated with PCI within the EXCEL [7], while only a minority of patients included in the NOBLE study were on ticagrelor and some even received ticlopidine in addition to ASA [8]. In addition, the differences between the use of DAPT in the included studies might have a differential impact depending on the diverse implantation techniques in single studies. In fact, as the treatment approach was left at the operators’ discretion, selected studies present some heterogeneities. Among the others, a higher use of culotte stenting in the NOBLE is clearly standing out. In this regard, the virtual absence of TIMI major or minor bleeding events beyond the first month and up to 3 years in the PCI arm of the EXCEL study suggests that DAPT prolongation and the use of newer P2Y12 antagonists after LMCA PCI is a safe strategy.

Although very recently some updated meta-analyses performed [20, 21], as the results of the NOBLE and EXCEL studies had become available, these were not focused on long-term outcomes. In fact, they also included 12-months follow up data [22]. In addition, none of them included results of the LE MANS study [18]. For this reason, this is the most comprehensive and updated meta-analysis comparing the long-term outcome after treatment of LMCA disease with CABG or Stent-PCI.

### Repeat revascularization (RR)

Results on the single endpoint of RR deserves some attention. The evaluation of RR in studies comparing Stent-PCI and CABG for LMCA revascularization has always been a debated issue, for several reasons. In fact, while some interpret repeat revascularization as a failure of the primary revascularization strategy, others claim that repeat revascularization is a surrogate endpoint for angina and shouldn’t be mixed up with harder clinical endpoint such as death, stroke or MI. This represents a key issue in study design, since inclusion of RR in the primary composite endpoint often yields quite different results. In fact, the rate of repeat revascularization was substantially higher in the PCI arm in almost all included studies, with the exception of the LE MANS study, the only one reporting a 10-years follow up, suggesting a late catch-up phenomenon for CABG towards PCI on a longer follow up [18]. Notwithstanding the still open debate on this issue, addition of RR to the composite efficacy endpoint in the present meta-analysis resulted in a significantly better outcome for CABG, although a large part of these events was related to de novo lesions and non LMCA target lesions. In fact, primary endpoint results of the NOBLE study were driven by the rate of repeat revascularization, which was higher in the PCI group (16% versus 10%, *p* = 0·032), especially on non-target lesions [7]. On the contrary, no difference was found in the rate of target LMCA revascularization (10% versus 9%, *p* = 0·37, Additional file 6: Table S1) [7].

### Risk stratification based on coronary anatomy

Patients’ stratification by means of the SS revealed superiority of CABG only within the subgroup of patients with a SS ≥33, with no difference between CABG and Stent-PCI for patients with a SS <33, despite addiction of RR to the composite endpoint. Further stratification showed no difference in both the low- (SS <23) and the intermediate-risk (SS 23–32) groups. These results are in line with previous data from both randomized and non-randomized studies, showing that CABG performs better with very complex or multivessel coronary artery disease [16]. In fact, presence of three vessel disease (3VD) in addition to significant LMCA disease was associated with better performance of CABG, compared to PCI in several studies. The reasons for these results are most probably related to the possibility to achieve more complete revascularization with CABG in similar cases. In fact, bypassing longer lesions is technically easier with CABG and seems to be more effective than with PCI, whereas implantation of longer or multiple stents can be associated to higher rates of restenosis and stent thrombosis. In line with this hypothesis, is has been reported that incomplete revascularization in patients with complex CAD more commonly occurs after Stent-PCI than with CABG [23, 24]. Nonetheless, differences in study design may have also had an impact. In fact, while patients with a SS above 32 were excluded from the EXCEL trial, the NOBLE used different upper cut off limit for CAD extension [7, 8].

### Risk stratification based on patients’ clinical condition

Results of the present meta-analysis suggest that in addition to the stratification based on coronary anatomy, some clinical variables have a relevant impact and should be taken into account when selecting the most appropriate revascularization strategy for individual patients. In fact, at meta-regression we found that a higher percentage of diabetics was associated to a better performance of CABG over PCI, confirming previous evidence on the negative impact of diabetes on clinical success after Stent-PCI. A novel finding of this meta-analysis was the evidence that CABG generally performs better in female patients. This is in line with the results of very recent studies on CABG, showing that the renowned “excess mortality” that had been traditionally observed for women undergoing CABG is indeed limited to the short-term or peri-procedural phase [25, 26]. On the contrary, a higher patients’ age was associated to a better performance of Stent-PCI over CABG. This is a relevant new observation, since previous meta-analyses had not sufficiently addressed the impact of patients’ age. On the contrary, this finding could have a useful clinical application in the implementation of patients’ stratification strategies. In fact, the addition of clinical parameters on top of anatomical scores could further improve selection of the best revascularization strategy in individual patients [24]. In addition, the experimental design of future studies addressing this clinical issue should take into accoun the impact of patients’ age.

### Implantation technique, device quality and operators’ proficiency

Also the implantation technique and operators’ proficiency may have an impact on patient outcomes after Stent-PCI of the LMCA [27, 28]. To this regard, newer studies have more stringent implantation protocols, which provides more accurate information to caring physicians [7, 8].

To this regard, it should be noted that the use of intracoronary imaging to guide PCI was not homogeneous between the studies. Even though the percetage use of these technique had no significant impact on the difference in the endpoints assessed between Stent-PCI and CABG, we cannot exclude a residual effect on procedure quality. However, operators’ proficiency is not the only relevant issue laying on the table. In fact, the use of contemporary DES, together with improvements in PCI technique, imaging guidance, physiological lesion assessment, as well as anti-platelet therapy, has brought about a substantial improvement in PCI outcomes, and allowed extension of PCI treatment options to increasingly complex patient and lesion subsets [4, 29]. In this regard, it is important to underline that a number of the patients included in the Stent-PCI arm of the present meta-analysis had been treated with 1st generation DES or even bare metal stents (BMS) [17, 19], while only the newly published trials used 2nd generation DES, also including biolimus-eluting stents. In this context, several non-randomized studies have suggested that 2nd generation everolimus-eluting stents contributed to the improvement in outcomes after PCI of LMCA disease [30, 31]. Hence, it is possible that a larger use of newer devices, implantation techniques and pharmacological therapies will allow a larger proportion of patients with significant LMCA disease to be treated with PCI in the future [32]. To this regard, a recent comprehensive cost-effectiveness analysis revealed that, although CABG shows a more favourable profile as compared to PCI, cost-effectiveness of percutaneous revascularization is better than CABG in patients with LMCA disease [33].

It is noteworthy to mention that a Heart Team approach is of key importance to achieve the most complete revascularization. In fact, routine patients’ evaluation by the heart team in the EXCEL study was associated to excellent results, both in the Stent-PCI and in the CABG arms. [7]

### Study limitations

Although no large heterogeneity was found between the randomized studies included in the present analysis, there were differences in study design, procedural characteristics and follow up lenght. In particular, the differences in the primary endpoint partly explain the heterogeneity in sample size between the studies. In addition, different stent types had been used in the selected studies, including BMS, 1st- and 2nd-generation DESs. Unfortunately, data on single stent types or even categories were not available from the selected studies. Hence, we cannot completely exclude a concealed impact of the varying use of different stent types on meta-analysis results. To account for these potential sources of heterogeneity, we used a random effects model for all analyses, as previously described [34]. Even though the present analysis only included high quality, randomized studies, some potential source for bias may still persist. For example, the study designs did not allow patients’ or investigators’ blinding. As described in the results section, technical execution of both CABG and PCI presents several differences between the studies. These are mostly related to the different times when single studies were run, even though differences in center-specific protocols also account for some degree of heterogeneity. In this regard, the newly published studies had more stringent protocols, to warrant homogeneous treatment across the studies [7, 8]. Despite a wide patients’ range was included in the selected studies, results of the present analysis may not apply to specific patients’ subsets. For example, patients with larger LMCA were excluded from most studies, due to unavailability of the study-specific DES for those diameters. At the same time, this could represent and additional concealed source for bias, since the LMCA diameter range to be eligible were not homogeneous across the studies. Finally, studies with larger proportions of diabetics had often included also a larger prevalence of 3VD, making difficult to distinguish the impact from these single moderators. Finally, detailed data on bleeding events are only available from the original studies, but not reported in all long-term studies included in this meta-analysis. For this reason, it was not possible to perform an analysis on this outcome.

## Conclusions

Results of the present meta-analysis demonstrate that similar outcomes are to be expected with Stent-PCI and CABG in appropriately selected patients with significant LMCA disease, when performed by experienced teams. Nevertheless, the shorter hospital stay, the better early safety profile, and the faster recovery after revascularization, and a more favourable cost-effectiveness make PCI very attractive. On the other hand, particular attention should be focused on patients’ selection, as this meta-analysis revealed a better performance for CABG in specific patients’ categories, such as more complex anatomical settings or female patients, whereas Stent-PCI shows a better performance in older age and less extensive coronary vascular disease.

## Additional files

Additional file 1: Supplementary material 1. PRISMA Statement. (DOC 63 kb)

Additional file 2: Figure S1. Risk of bias. Summary of the study quality analysis. (PDF 773 kb)

Additional file 3: Figure S2. Funnel plots. Panel A. Funnel plot for the composite endpoint of death, myocardial infarction (MI) and stroke, demonstrating no evidence of publication bias. Panel B. Funnel plot for the composite endpoint of death, myocardial infarction (MI), stroke anr repeat revascularization (RR), demonstrating no evidence of publication bias. Each circle represents a study. Study precision (reported on the y-axis as the Standard Error of the Log OR) is plottet against the summary effect. (PDF 293 kb)

Additional file 4: Figure S3. The role of different follow-up on primary composite endpoint (Death, MI, Stroke). (PPT 146 kb)

Additional file 5: Supplementary Material 5. Endpoint definitions. (DOCX 145 kb)

Additional file 6: Table S1. TVF and TLF. (DOCX 14 kb)
