# Supplementary material for: Long-term outcomes of coronary artery bypass grafting versus stent-PCI for unprotected left main disease: a meta-analysis
Source: BMC Cardiovasc Disord. 2017 Sep 6;17:240. doi: 10.1186/s12872-017-0664-5 (PMC5588710; doi:10.1186/s12872-017-0664-5)
Supplement: Supplementary file 2 — Risk of bias. Summary of the study quality analysis. (PDF 773 kb) [file 12872_2017_664_MOESM2_ESM.pdf]

	Random sequence generation (selection bias)	Allocation concealment (selection bias)	Blinding (performance bias and detection bias)	Incomplete outcome data (attrition bias)	Selective reporting (reporting bias)	Other bias
**EXCEL 2016**	✓	✓	✗	✓	✓	✓
**NOBLE 2016**	✓	✓	✗	✓	✓	✓
**LE MANS 2016**	✓	✓	✗	✓	✓	✓
**PRECOMBAT 2015**	✓	✓	✗	✓	✓	✓
**SYNTAX 2014**	✓	✓	✗	✓	✓	✓
