# Supplementary figures and images for: Long-term outcomes of coronary artery bypass grafting versus stent-PCI for unprotected left main disease: a meta-analysis
Source: BMC Cardiovasc Disord. 2017 Sep 6;17:240. doi: 10.1186/s12872-017-0664-5 (PMC5588710; doi:10.1186/s12872-017-0664-5)

**A**

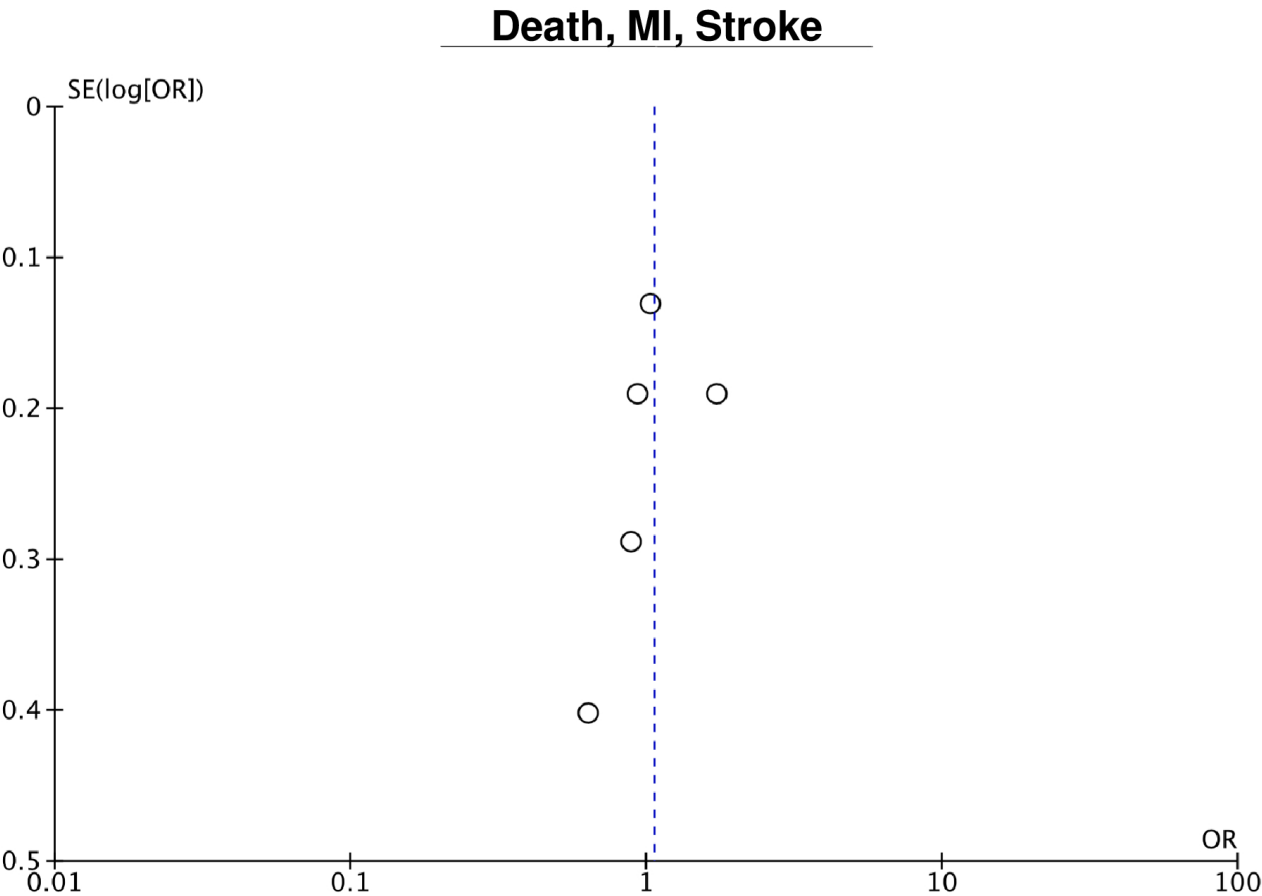

**B**

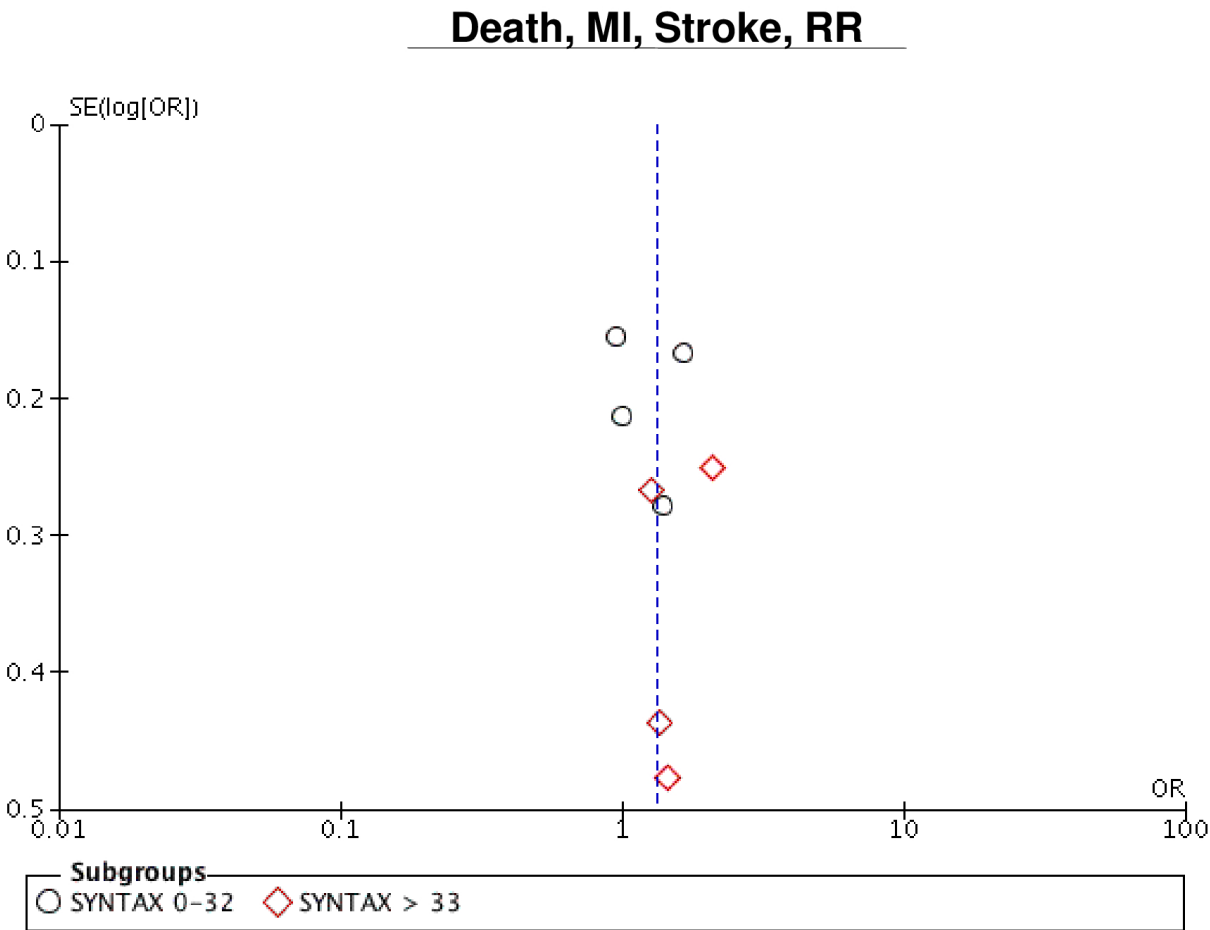

Supplement: Supplementary file 3 — Funnel plots. Panel A. Funnel plot for the composite endpoint of death, myocardial infarction (MI) and stroke, demonstrating no evidence of publication bias. Panel B. Funnel plot for the composite endpoint of death, myocardial infarction (MI), stroke anr repeat revascularization (RR), demonstrating no evidence of publication bias. Each circle represents a study. Study precision (reported on the y-axis as the Standard Error of the Log OR) is plottet against the summary effect. (PDF 293 kb) [file 12872_2017_664_MOESM3_ESM.pdf]

## Slide 1
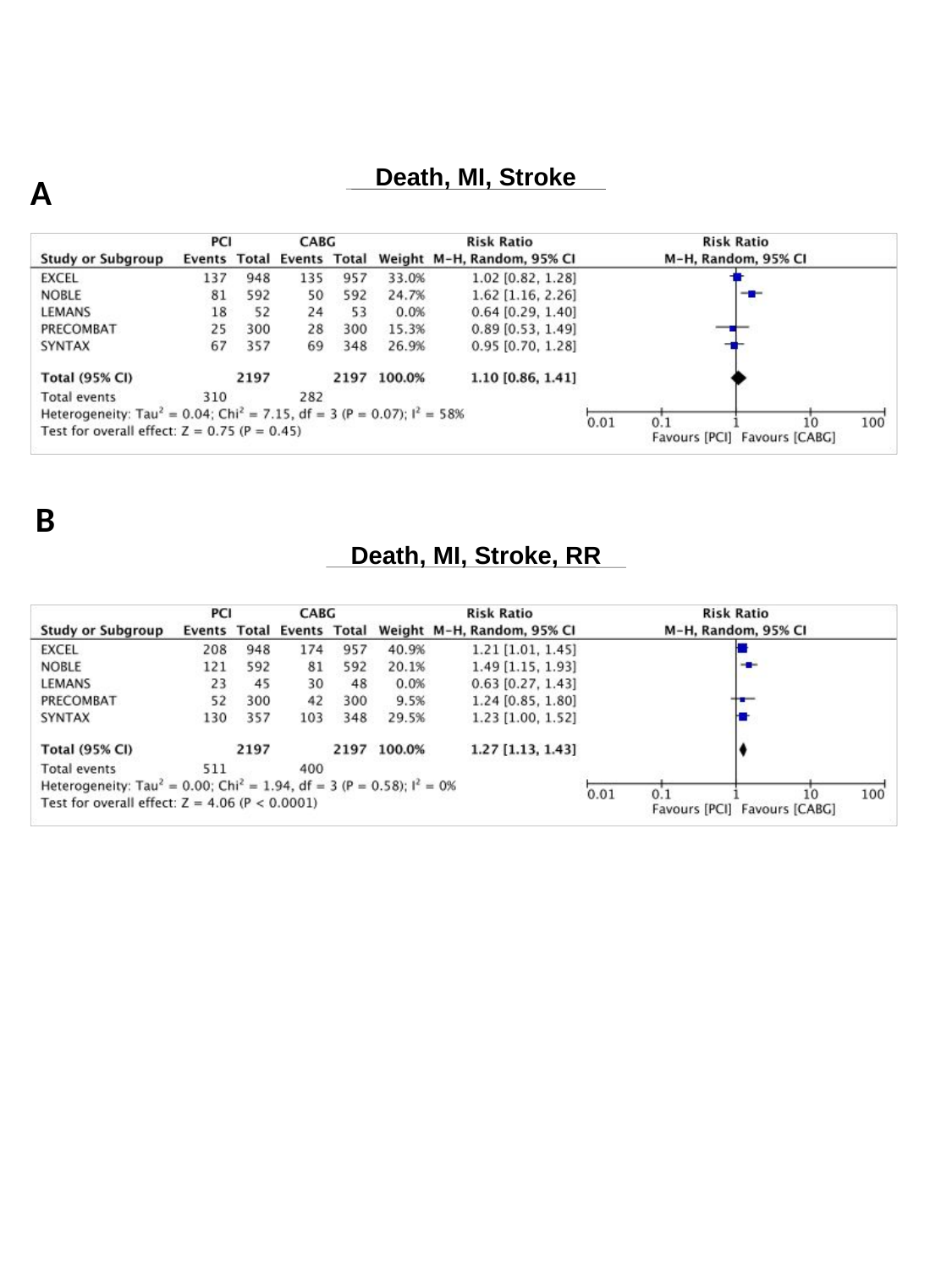

A
Death, MI, Stroke
B
Death, MI, Stroke, RR

Supplement: Supplementary file 4 — The role of different follow-up on primary composite endpoint (Death, MI, Stroke). (PPT 146 kb) [file 12872_2017_664_MOESM4_ESM.ppt]
