# Supplementary material for: Long-term outcomes of coronary artery bypass grafting versus stent-PCI for unprotected left main disease: a meta-analysis
Source: BMC Cardiovasc Disord. 2017 Sep 6;17:240. doi: 10.1186/s12872-017-0664-5 (PMC5588710; doi:10.1186/s12872-017-0664-5)
Supplement: Supplementary file 5 — Endpoint definitions. (DOCX 145 kb) [file 12872_2017_664_MOESM5_ESM.docx]

**Endpoint definitions**

**EXCEL**

**Death**

The cause of death will be adjudicated as being due to cardiovascular causes, non-cardiovascular causes, or undetermined causes.

• Cardiovascular death includes sudden cardiac death, death due to acute MI, heart failure or cardiogenic shock, stroke, other cardiovascular causes, or bleeding

• Non-cardiovascular death is defined as any death with known cause not of cardiac or vascular causes

• Undetermined cause of death refers to a death not attributable to one of the above categories of cardiovascular death or to a noncardiovascular cause. For this trial all deaths of undetermined cause will be included in the cardiovascular category

**Myocardial infarction (protocol definition)**

**Post procedure MI:** Defined as the occurrence within 72 hours after either PCI or CABG of either:

• CK-MB >10x upper reference limit (URL)*, OR

• CK-MB >5x URL*, PLUS

- new pathological Q waves in at least 2 contiguous leads or new persistent non-rate related LBBB, or

- angiographically documented graft or native coronary artery occlusion or new severe stenosis with thrombosis and/or diminished epicardial flow, or

- imaging evidence of new loss of viable myocardium or new regional wall motion abnormality

**Spontaneous MI:** defined as the occurrence >72 hours after any PCI or CABG of:

• The rise and/or fall of cardiac biomarkers (CK-MB or troponin) >1x URL* PLUS:

- ECG changes indicative of new ischemia [ST-segment elevation or depression, in the absence of other causes of ST-segment changes such as left ventricular hypertrophy (LVH) or bundle branch block (BBB)], or

- Development of pathological Q waves ( ≥0.04 seconds in duration and ≥1 mm in depth) in ≥2 contiguous precordial leads or ≥2 adjacent limb leads) of the ECG, or

- Angiographically documented graft or native coronary artery occlusion or new severe stenosis with thrombosis and/or diminished epicardial flow, or

- Imaging evidence of new loss of viable myocardium or new regional wall motion abnormality

Each MI will also be adjudicated as:

• ST-segment elevation MI (STEMI)

• Non-ST-segment elevation MI (NSTEMI)

• Each STEMI and NSTEMI will be subcategorized as

- Q-wave

- Non-Q-wave

- Unknown (no ECG or ECG not interpretable)

**Stroke**

The rapid onset of a new persistent neurologic deficit attributed to an obstruction in cerebral blood flow and/or cerebral hemorrhage with no apparent non-vascular cause (e.g., trauma, tumor, or infection). A vascular neurologist or stroke specialist will determine whether a stroke has occurred and determine the stroke severity using the NIHSS TIA/Stroke

questionnaire. Available neuroimaging studies will be considered to support the clinical impression and to determine if there is a demonstrable lesion compatible with an acute stroke. Strokes will be classified as ischemic, hemorrhagic, or unknown. Four criteria must be fulfilled to diagnosis stroke:

1. Rapid onset of a focal/global neurological deficit with at least one of the following: change in level of consciousness, hemiplegia, hemiparesis, numbness or sensory loss affecting one side of the body, dysphasia/aphasia, hemianopia, amaurosis fugax, other new neurological sign(s)/symptom(s) consistent with stroke; and

2. Duration of a focal/global neurological deficit ≥24 hours or <24 hours if any of the following conditions exist:i. At least one of the following therapeutic interventions: a. Pharmacologic (i.e., thrombolytic drug administration) b. Non pharmacologic (i.e., neurointerventional procedure such as intracranial angioplasty) ii. Available brain imaging clearly documents a new hemorrhage or infarct iii. The neurological deficit results in death

3. No other readily identifiable non-stroke cause for the clinical presentation (e.g., brain tumor, trauma, infection, hypoglycemia, other metabolic abnormality, peripheral lesion, or drug side effect). Patients with non-focal global encephalopathy will not be reported as a stroke without unequivocal evidence based upon neuroimaging

studies.

4. Confirmation of the diagnosis by a neurology or neurosurgical specialist and at least one of the following:

a. Brain imaging procedure (at least one of the following):

i. CT scan

ii. MRI scan

iii. Cerebral vessel angiography

b. Lumbar puncture (i.e. spinal fluid analysis diagnostic of intracranial hemorrhage) All strokes with stroke disability of modified Rankin Scale (mRS) ≥1 (increase from baseline assessment) will be included in the primary endpoint. All diagnosed strokes (even with mRS 0) will also be tabulated.

**Ischemia-driven revascularization**

A coronary revascularization procedure may be either a CABG or a PCI. The coronary segments revascularized will be sub-classified as:

• Target Lesion: A lesion revascularized in the index procedure (or during a planned or provisional staged procedure). The LM target lesion extends from the left main stem ostium to the end of the 5 mm proximal segments of the left anterior descending and left circumflex arteries as well as the ramus intermedius if the latter vessel has a vessel diameter of ≥2 mm.

• Target Vessel: The target vessel is defined as the entire major coronary vessel proximal and distal to the target lesion including upstream and downstream branches and the target lesion itself. The left main and any vessel originating from the left main coronary artery or its major branches is, by definition, considered a target vessel for the purposes of this trial (unless either the LAD or LCX are occluded at baseline and no attempt was made to revascularize these territories by either PCI or CABG).

• Target Vessel Non-Target Lesion: The target vessel non-target lesion consists of a lesion in the epicardial vessel/branch/graft that contains the target lesion; however, this lesion is outside of the target lesion by at least 5 mm distal or proximal to the target lesion determined by quantitative coronary angiography (QCA).

• Non-Target Vessel: For the purposes of this trial, the only possible non-target vessel would be the right coronary artery and its major branches that were not treated by either PCI or CABG at the index procedure (unless either the LAD or LCX are occluded at baseline and no attempt was made to revascularize these territories by either PCI or CABG). All revascularization events will be adjudicated as either ischemia-driven or non-ischemia-driven. Revascularization will be considered ischemiadriven if the diameter stenosis of the revascularized coronary segment is ≥50% by QCA and any of the following criteria for ischemia are met:

• A positive functional study corresponding to the area served by the target lesion; or

• Ischemic ECG changes at rest in a distribution consistent with the target vessel; or

• Typical ischemic symptoms referable to the target lesion; or • IVUS of the target lesion with a minimal lumen area (MLA) of ≤4 mm2 for non-left main lesions or ≤6 mm2 for left main lesions. If the lesions are de novo (i.e. not restenotic), the plaque burden must also be ≥60%; or

• FFR of the target lesion ≤0.80

A target lesion revascularization for a diameter stenosis less than 50% might also be considered ischemia-driven by the Clinical Events Committee if there was a markedly positive functional study or ECG changes corresponding to the area served by the target lesion.

**Peri-procedural major adverse events****

The composite rate of any of the following, occurring within 30-days post procedure

• Death

• Stroke

• Myocardial infarction

• Ischemia-driven revascularization

• TIMI major or minor bleeding

• Transfusion ≥2 units of blood

• Major arrhythmia (supraventricular tachycardia requiring cardioversion, ventricular tachycardia or fibrillation requiring treatment, or bradyarrhythmia requiring temporary or permanent pacemaker)

• Any unplanned surgery or therapeutic radiologic procedure

• Renal failure (serum creatinine increase by ≥0.5 mg/dL from baseline

or need for dialysis)

• Sternal wound dehiscence

• Infection requiring antibiotics

• Prolonged intubation (>48 hours)

• Post-pericardiotomy syndrome

*Local laboratory upper limit of normal (ULN) will be used if the URL is not available. **Death, stroke, myocardial infarction, and ischemia-driven revascularization are adjudicated by the clinical events committee; the remainder of peri-procedural events are site reported.

**NOBLE**

**All–cause mortality**

Death from any cause.

**Cardiac death**

Cardiac death was defined as any death due to a suspected cardiac cause (myocardial infarction, low–output 145 heart failure, fatal arrhythmia), unwitnessed death and death of unknown cause. All procedure–related 146 deaths, including those related to concomitant treatment, were classified as cardiac death. The endpoint was 147 included post hoc. (Modified from Cutlip et al. Circulation. 2007;115:2344–2351) The information on cause 148 of death was obtained from hospital patient files, from general practitioners, or from families if no other 149 source was available. 150

**Non–procedure–related myocardial infarction**

A rise in biochemical markers exceeding the decision limit for myocardial infarction (99th percentile 158 including < 10% CV) with at least one of the following; (1) ischemic symptoms, (2) ECG changes indicative 159 of ischemia (ST segment elevation or depression), and (3) development of a pathologic Q–wave with no 160 relation to a PCI procedure. 161

**Repeat revascularisation**

Any new PCI or CABG operation performed during follow–up. If an index revascularisation was attempted 164 or successful, any subsequent revascularisation was counted as repeat revascularisation. Attempted PCI was 165 defined as an advancement of a wire in the coronary tree at least. Attempted CABG was defined as at least 166 initiation of an index operation. 167

**Procedure–related biomarker release**

The diagnosis of a procedure–related biomarker increase required a rise in total creatine kinase (CK) and/or 170 Troponin–T/I. Due to the great heterogeneity of biomarkers and various assays used during the study in 171 participating centres, this comparison was omitted from the final analysis.

**Procedural myocardial infarction**

Diagnosis of procedural MI for both PCI and CABG patients was based on CK–MB elevations when 176 available. Patients needed to have stable angina pectoris as the clinical indication OR a normal baseline CK–177 MB, TnI, TnT, or highly sensitive TnT, to be assessable for procedural MI. Diagnosis required a CK–MB 178 value above 10 x URL or ULN to establish the diagnosis. The diagnosis could also be placed by the 179 combination of a CK–MB value above 5 x URL or ULN, AND one or more of the following: (1) new 180 pathological Q waves in at least 2 contiguous leads or new persistent non–rate–related left bundle branch 181 block, or (2) angiographically documented graft or native coronary artery occlusion or new severe stenosis 182 with thrombosis and/or diminished epicardial flow, or (3) imaging evidence of new loss of viable 183 myocardium or new regional wall motion abnormality. The endpoint of procedural myocardial infarction 184 was included post hoc and the definition was adapted to match the definition applied in the EXCEL trial on 185 PCI vs. CABG for LMCA stenosis. Peri-procedural MI due to repeat revascularization during follow-up 186 were assessed applying the 3rd Universal definition as CK-MB was not available in all event patients. A 187 procedural MI according to this definition was counted as a non-index procedural myocardial infarction.

**Target lesion revascularisation**

Repeat revascularisation by PCI of any target segment treated during the index procedure. A target lesion 192 segment was defined as a stented or balloon treated segment and its 5 mm margins.

**SYNTAX**

Deaths were considered cardiac unless an unequivocal, noncardiac cause was established. CVA was defined as a focal, central neurological deficit lasting >72 hours (h) which resulted in irreversible brain damage or body impairment. Repeat

revascularization was defined as any repeat PCI or CABG. Complete revascularization was defined as the successful treatment of all eligible lesions identified during the Heart Team conference and estimated post procedure by the investigator. The definition of MI was based on previous studies42, 43 MI was defined in relation to intervention status as follows i) after allocation but before treatment: Q-wave (new pathological Q-waves in ≥2 leads lasting ≥0.04 seconds with CK-MB levels elevated above normal), and non-Qwave MI (elevation of CK levels >2 times the upper limit of normal [ULN] with positive CK-MB or elevation of CK levels to >2 times ULN without new Q-waves if no baseline CK-MB was available); ii) <7d after intervention: new Q-waves and either peak CKOne- year outcomes of the SYNTAX trial MB/total CK >10% or plasma level of CK-MB 5x ULN; iii) ≥7d after intervention: new Qwaves or peak CK-MB/total CK >10% or plasma level of CK-MB 5x ULN or plasma level of CK 5x ULN. The CK/CK-MB enzyme levels were obtained and measured by a core laboratory for all randomized patients. Per protocol graft occlusion (GO) and stent thrombosis (ST) were considered acute if occurring ≤24h following the study procedure, sub-acute if occurring >24h to ≤30d following the study procedure and late after 30d. Per protocol graft occlusion and stent thrombosis were defined as either: i) clinical presentation of an acute coronary syndrome with documentation of a flow limiting thrombus or occlusion within a bypass graft or adjacent to the anastomosis of a previously bypassed coronary artery (for CABG patients) or within or adjacent to a previously successfully treated artery (for PCI patients); ii) a Q-wave MI in the territory of ≥1 treated vessels within first 30 days (d). Secondary Endpoints Additional endpoints include overall MACCE at 1 month postprocedure, and at 6 months, 3 and 5 years post-allocation as well as the rates of the individual components of MACCE at the same time points as overall MACCE. Quality of life at 1 month postprocedure, and at 6 months, 1, 3 and 5 years post-allocation and cost and costeffectiveness at 1, 3 and 5 years post-allocation were additional prespecified secondary endpoints, as was the SYNTAX Score.

**LE MANS**

Secondary end points. Secondary end points included 30-day and 1-year MAE and MACCE, length of hospitalization, exercise tolerance measured with an electrocardiographic treadmill stress test along with angina severity according to the Canadian Cardiovascular Society classification after 1 year, total survival and freedom from MACCE, and target vessel failure (TVF) and revascularization (TVR). The MAE were defined as all-cause mortality, acute myocardial infarction (defined as an increase in creatine phosphokinase (CPK)-MB to higher than 3 times the upper limit of normal after PCI and 5 times after CABG), repeat revascularization, acute heart failure (e.g., pulmonary edema, cardiogenic shock), or low output syndrome requiring intravenous inotropic agents and/or intra-aortic balloon pump support, post-procedural complications leading to reintervention, stroke, arrhythmia (ventricular fibrillation, ventricular tachycardia, or atrial fibrillation), major bleeding requiring additional blood transfusion, and infections compromising post-procedural rehabilitation. Any cardiac mortality, acute myocardial infarction, stroke, repeat intervention, and/or acute/subacute in-stent thrombosis were considered MACCE. Target vessel failure was defined as

any MACCE related to insufficient flow through the LMCA, and TVR as any repeat intervention (PCI or CABG) caused by a narrowing of the LMCA. The incidence of stent thrombosis was evaluated in accordance with the Academic Research Consortium Definitions of Stent Thrombosis.

**PRECOMBAT**

Definitions of End Points

Deaths were considered cardiac unless an unequivocal, noncardiac cause was established. MI was defined as new Q waves and increase in the creatine kinase MB concentration to greater than five times the upper limit of the normal range, if occurring within 48 hours after the procedure, or as new Q waves or an increase in creatine kinase MB concentration to greater than the upper limit of the normal range, plus ischemic symptoms or signs, if occurring more than 48 hours after the procedure. Stroke was defined as a sudden onset of vertigo, numbness, aphasia, or dysarthria resulting from vascular lesions of the brain, including hemorrhage, embolism, thrombosis, or rupturing aneurysm, and persisting for >24 hours. Protocol-defined graft occlusion or stent thrombosis was defined as clinical presentation of an acute coronary syndrome with documentation of flow limiting thrombus or occlusion within a bypass graft or adjacent to the anastomosis of a previously bypassed coronary artery (for CABG patients),

or within or adjacent to a previously successfully treated artery (for PCI patients), or as a Q wave in the territory of ≥ 1 treated vessels within 30 days.3 Target vessel revascularization (TVR), in which repeat revascularization with either PCI or CABG was performed in the treated vessel, was considered to be driven by ischemia if the stenosis of any vessel was at least 50% of the diameter of the vessel in the presence of ischemic signs or symptoms or if the stenosis was at least 70% of the diameter of the vessel even in the absence of ischemic signs or symptoms. Alternatively, TVR was considered clinically driven when the treated vessels had stenosis of at least 50% in the presence of ischemic signs or symptoms. In addition, any revascularization was defined as revascularization in any vessel during follow- up. Crossover was defined as completion of the procedure opposite the one designated by the randomization scheme within 30 days after randomization.
