# Supplementary material for: Long-term outcomes of coronary artery bypass grafting versus stent-PCI for unprotected left main disease: a meta-analysis
Source: BMC Cardiovasc Disord. 2017 Sep 6;17:240. doi: 10.1186/s12872-017-0664-5 (PMC5588710; doi:10.1186/s12872-017-0664-5)
Supplement: Supplementary file 6 — TVF and TLF. (DOCX 14 kb) [file 12872_2017_664_MOESM6_ESM.docx]

**Table S1**

|  | **SYNTAX**  **2014** | | **PRECOMBAT**  **2015** | | **LE MANS**  **2016** | | **NOBLE**  **2016** | | **EXCEL**  **2016** | | |
| --- | --- | --- | --- | --- | --- | --- | --- | --- | --- | --- | --- |
|  | PCI | CABG | PCI | CABG | PCI | CABG | PCI | CABG | PCI | CABG |  |
| **Target Vessel Failure, %** | - | - | 12.4 | 6.3 | - | - | 10 | 9 | 10.9 | 7.2 |  |
| **Target Lesion Failure, %** | - | - | - | - | - | - | 12 | 8 | 9.5 | 6.9 |  |

PCI= percutaneous coronary intervention; CABG= coronary artery bypass grafting
